# Successful delivery in a patient with ovarian hyperstimulation syndrome complicated by deep vein thrombosis: A case report

**DOI:** 10.1097/MD.0000000000043187

**Published:** 2025-07-11

**Authors:** Aike Xu, Linfang Zhao, Qun Wei

**Affiliations:** a Reproductive Center, Sir Run Run Shaw Hospital, Zhejiang University School of Medicine, Hangzhou, Zhejiang, China; b Nursing Department, Sir Run Run Shaw Hospital Zhejiang University School of Medicine, Hangzhou, Zhejiang, China.

**Keywords:** controlled ovulation induction, deep vein thrombosis, ovarian hyperstimulation syndrome, Self-Care Theory, shared decision-making

## Abstract

**Rationale:**

In this study, we aimed to enhance the self-care abilities of a patient with severe ovarian hyperstimulation syndrome complicated by multiple thromboses in the muscular veins of the left lower extremity, guided by Orem’s Self-Care Theory.

**Patient concerns:**

When does ovarian hyperstimulation syndrome subside; whether venous thromboembolism be cured; whether there will be sequelae; when can frozen-thawed embryo transfer be done; when can one get pregnant.

**Diagnoses:**

Severe ovarian hyperstimulation syndrome complicated by multiple thromboses in the intermuscular vein of the left lower extremity.

**Interventions:**

Using a supportive educational nursing system, the patient successfully performed self-assessment during the acute phase of venous thromboembolism and actively participated in adjusting and implementing the nursing intervention plan. Continuous monitoring of the patient’s condition was ensured through follow-up, dynamic assessment, and ongoing adaptation of personalized support and education. Furthermore, a shared decision-making approach was used to formulate a management plan aimed at preventing the recurrence of thrombosis post-transplantation.

**Outcomes:**

Following effective treatment and nursing interventions, the patient achieved a successful pregnancy and delivery, with no recurrence of thrombosis.

**Lessons:**

The application of Orem’s Self-Care Theory in nursing practice for patients with severe ovarian hyperstimulation syndrome complicated by deep vein thrombosis can improve patient participation, satisfaction, and pregnancy outcomes, and reduce the occurrence of nursing errors and accidents.

## 1. Introduction

Ovarian hyperstimulation syndrome (OHSS) is a common iatrogenic complication associated with controlled ovarian hyperstimulation (COH) treatment. Severe cases of OHSS can result in life-threatening symptoms, including pleural effusion, acute renal insufficiency, and venous thromboembolism (VTE).^[1]^ The incidence of OHSS (mild, moderate and severe) in patients with polycystic ovary syndrome (PCOS) has been estimated to be as high as 17% to 31%, affecting women undergoing fertility treatment.^[1]^ The pooled frequency of VTE associated with assisted reproductive technology (ART) complicated by OHSS is <0.001% and the VTE risk is higher than that of ART without OHSS, despite a nonsignificant result.^[2]^ Once it occurs, severe OHSS can prove life-threatening. ART is characterized by long treatment cycles and complex medication regimens, making patients’self-care ability crucial to treatment outcomes. Orem Self-Care Theory emphasizes the importance of patients actively participating in their own care. Nurses play a vital role in transmitting health knowledge, providing skills training, offering counseling through education, and delivering emotional and material support to enhance patients’ active management of their own health. This approach encourages patients to participate in self-care and continuously improve their self-care abilities, contributing positively to assisted reproductive nursing practices.^[3]^ In this paper, we report a case involving an older primipara who developed severe OHSS complicated by multiple thromboses in the intermuscular vein of the left lower extremity during COH treatment. Guided by a framework based on Orem Self-Care Theory, early detection and intervention were implemented to actively prevent the recurrence of perinatal thrombosis. Ultimately, the patient delivered and navigated the postpartum period successfully.

## 2. Clinical data

### 2.1. General information

The patient was a 37-year-old woman with a 10-year history of primary infertility owing to PCOS. After 3 unsuccessful attempts at COH-assisted pregnancy at other facilities, she underwent in vitro fertilization and embryo transfer at our hospital in 2021. Following 2 months of oral contraceptive use, COH commenced with 100 IU/day of follicle-stimulating hormone β needle. Despite one-on-one medication guidance from a nurse, the patient failed to refrigerate her medication as instructed. The nurse emphasized the importance of correct medication storage and provided health education to prevent OHSS. The patient’s B-ultrasound showed a significant increase in bilateral ovarian volume. On the 11th day of COH, she received 3000 IU of intramuscular chorionic gonadotropin and 0.2 mg of subcutaneous triptorelin for triggering.

### 2.2. Treatment process and outcome (Fig. 1)

On the trigger day, the patient exhibited moderate depression edema in her left lower limb but reported no pain. After reporting this to the doctor and ruling out thrombosis, she was advised to wear elastic sockings, increase her fluid intake, and avoid prolonged sitting, and she received education on VTE prevention. Twelve hours after the trigger, she reported swelling and pain in the left leg, with laboratory results indicating elevated estrogen (>5286.00 pg/mL), progesterone (16.22 µg/L), luteinizing hormone (36.47 IU/L), and human chorionic gonadotropin (90.40 IU/L). Venous duplex ultrasonography (VDUS) (Fig. 2) confirmed intermuscular venous thrombosis in the left lower limb.^[4]^ A multidisciplinary team was assembled, and the patient was started on subcutaneous low-molecular-weight heparin (LMWH) injections twice daily and encouraged to walk more. Painless oocyte retrieval was performed under general anesthesia 12 hours after initiating LMWH, resulting in the collection of 34 oocytes. The patient was admitted to the hospital after the procedure, then received multidisciplinary consultation, fluid infusion, and anticoagulant therapy. Special attention was needed regarding vaginal bleeding, the left lower limb brakes, and the right lower limb moved properly. On the 4th postoperative day, the patient reported abdominal distension and blurred vision; relevant examinations were conducted, prompting an ophthalmology consult. Examination also revealed abdominal and bilateral thoracic effusions. By the 7th postoperative day, she experienced pain in her left leg, VDUS (Fig. 3) confirmed multiple thromboses in the intermuscular veins of the left lower leg. Anticoagulant therapy was continued without thrombolytic treatment. By the 12th postoperative day, the patient’s symptoms improved following abdominal drainage and ongoing anticoagulant therapy (Fig. 4). She was discharged per her physician’s advice, continuing anticoagulant treatment at home with regular vascular surgery follow-ups. Two months post-discharge, her anticoagulation treatment was adjusted owing to abnormal vaginal bleeding. Sixteen months later, she returned for frozen-thawed embryo transfer (FET), with successful implantation of a 4BB 2PN blastocyst. On the 10th day posttransfer, creatinine was measured at 80 µmol/L, and human chorionic gonadotropin was 449.6 IU/L. However, on the 26th day posttransfer, the patient exhibited significant bruising at the injection site and gingival bleeding. The D-dimer (DDi) value was 0.21 μg/mL, so LMWH was stopped per her doctor’s advice, and she was started on aspirin enteric-coated tablets (75 mg/d orally), which was discontinued at 12 weeks of pregnancy after examinations normal. By the end of 2023, the patient had delivered a healthy daughter weighing 3350 g at full term. Follow-up at 6 months postpartum revealed no recurrence of VTE.

**Figure 1.**
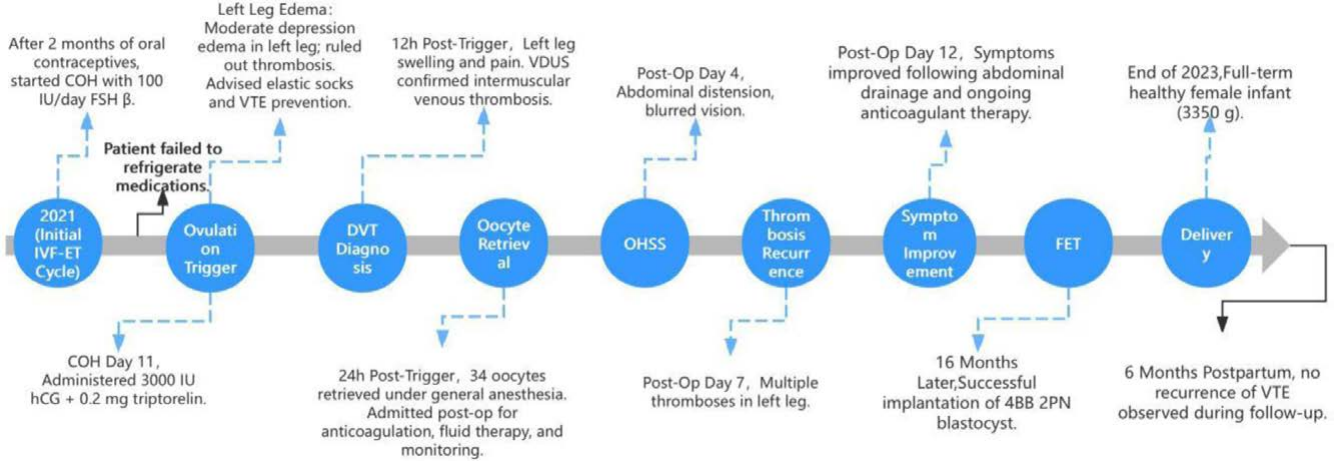
Timeline of events. COH = controlled ovarian hyperstimulation, DVT = deep vein thrombosis, FET = frozen-thawed embryo transfer, IVF-ET = in vitro fertilization and embryo transfer, LMWH = low-molecular-weight heparin, OHSS = ovarian hyperstimulation syndrome, VDUS = venous duplex ultrasonography, VTE = venous thromboembolism.

**Figure 2.**
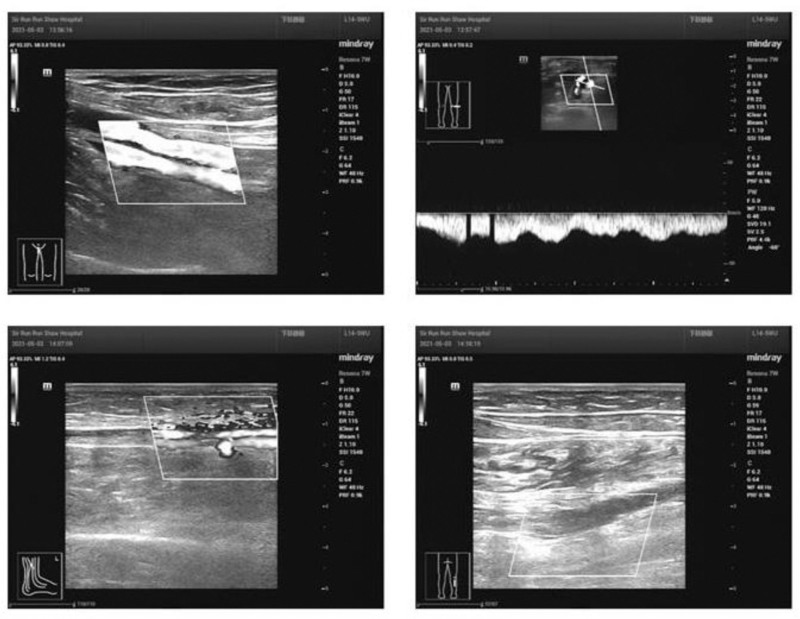
Venous duplex ultrasonography confirming lower extremity venous intermuscular thrombosis.

**Figure 3.**
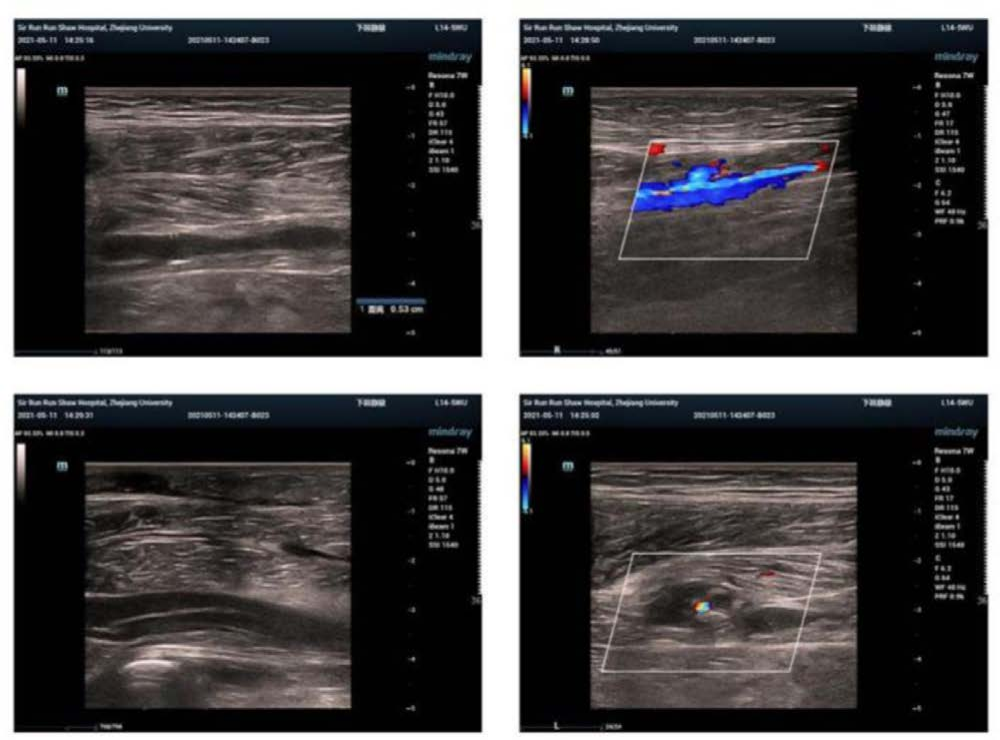
Venous duplex ultrasonography confirmed multiple thrombosis of the left lower extremity intermuscular vein.

**Figure 4.**
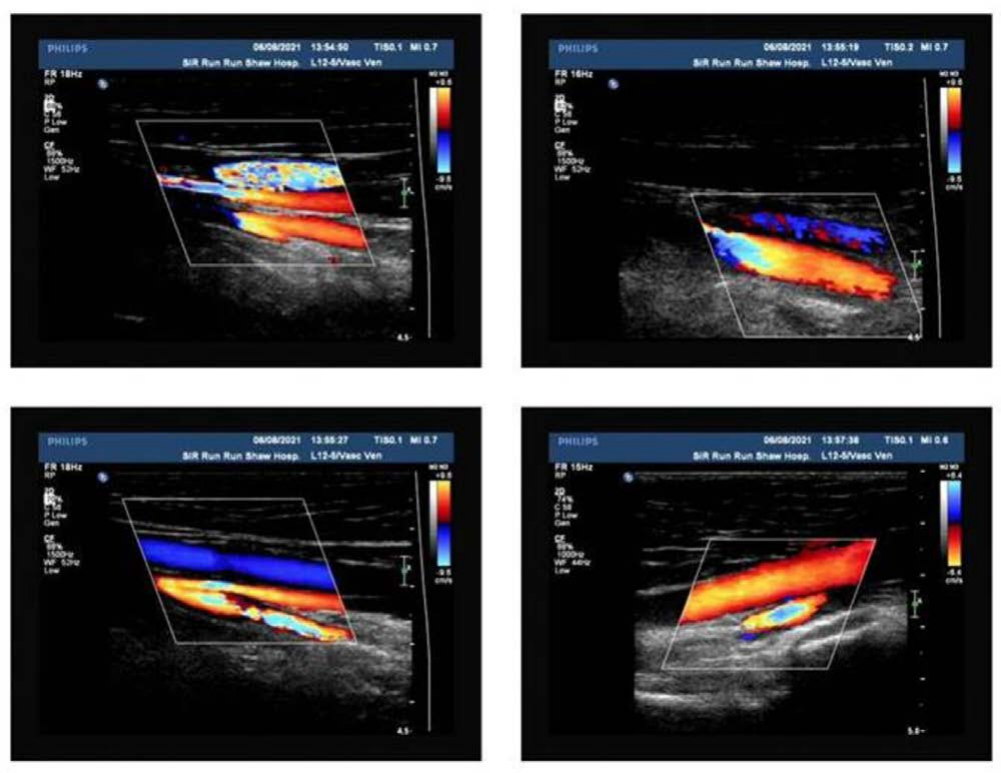
Dual-function ultrasound examination of the veins confirmed that the blood flow of the deep veins of both lower limbs was unobstructed.

## 3. Nursing

### 3.1. Identifying risks in the acute phase and adjusting the nursing intervention plan

#### 3.1.1. Tailoring health education based on patient profile according to thrombosis risk

**Figure 5.**
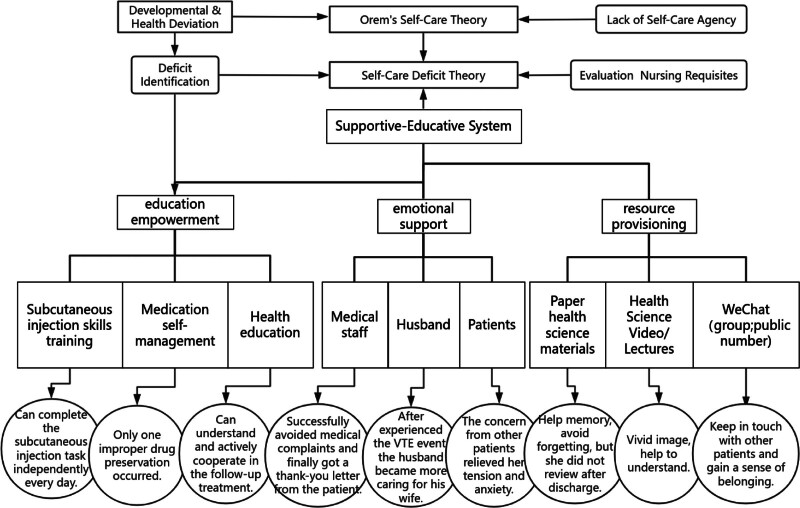
The framework of Orem Self-Care Deficit Nursing Theory applied in this patient. VTE = venous thromboembolism.

The patient, a junior college graduate and meditation coach, had previously experienced 3 unsuccessful treatments for ovulation induction at other hospitals. She exhibited clear subjective validation, poor compliance, and non-adherence behaviors, including unauthorized medication adjustments, rescheduling visits, and improper drug storage. Through face-to-face interviews, nurses thoroughly evaluated the patient’s medical knowledge level, learning capacity, and psychological state, to clarify her understanding of her condition and nursing knowledge as well as her information needs. Nurses found that the patient did not trust medical staff owing to the short outpatient consultation time, the doctor’s unilateral decision-making, and her previous failed medical experiences. A senior specialist nurse was arranged to be responsible for the patient and to develop personalized health education content for the patient according to the results of her evaluation (Fig. 5). This nurse guided the patient to actively participate in self-evaluation and to accurately record daily symptom changes, drug reactions, and physical conditions such as the degree of swelling in the left lower limb, pain score, and changes in skin color or temperature among others.^[5]^ The nurse also explained to the patient the importance of this information in managing her condition Because the patient had PCOS, she took short-acting contraceptives orally for 2 months before COH treatment. B-ultrasound showed more follicular development and high estrogen levels in both ovaries, which are high-risk factors for OHSS.^[1]^ When creating a nursing plan, the wishes of patient should be fully respected, and she was encouraged to participate in the decision-making process. In this case, face-to-face health education to prevent OHSS was strengthened and combined with paper informational materials and popular science videos. The teach-back technique was used to evaluate the patient’s mastery of OHSS.^[6,7]^ On the trigger day, the patient proactively reported foot swelling, so that her nurse could evaluate her and intervene promptly. Patient health education regarding OHSS-related complications was increased, especially symptom identification and coping strategies for VTE. This effectively enhanced the patient’s VTE risk awareness, and she returned to the hospital at the first experience of leg pain on the second day.

#### 3.1.2. Multidisciplinary team for perioperative management^[4]^

Prior to oocyte retrieval, the patient was diagnosed with acute distal deep vein thrombosis (DVT) and was assessed as having a low-risk for pulmonary embolism according to the Caprini scoring system. Mechanical intervention was prioritized for prevention. A multidisciplinary team was formed, comprising specialists in reproductive medicine, hematology, vascular surgery, respiratory medicine, assisted reproductive nursing, and anesthesiology. Nursing interventions were focused on supportive education and active care, incorporating direct patient communication via WeChat for ongoing engagement. The effectiveness of nursing strategies was dynamically evaluated, with particular attention paid to the patient’s emotional needs. LMWH was discontinued 12 hours before surgery to assess and reassess her bleeding risks.^[5]^ Routine care included encouraging the patient to consume 2000 to 2500 mL of water daily and adhered to a high-protein and vitamin-rich diet, promoting ambulation while avoiding strenuous activities or sudden positional changes, ensuring proper use of antithrombotic stockings, and encouraging the patient’s husband to accompany and support her emotionally.^[8]^ Health education on the mechanical and pharmacological prevention of VTE was reinforced. Intraoperative monitoring was focused on detecting bleeding complications. Postoperatively, the patient was rehydrated immediately upon admission, with anticoagulant therapy resumed 12 hours later. Additionally, she was advised to drink more water, initiate early bedside activities, and monitor herself closely for vaginal bleeding, hematuria, or anuria. Anticoagulant management was executed at critical intervals-preoperatively, postoperatively, and upon admission-with continuous and dynamic risk assessment implemented throughout the care process.

#### 3.1.3. Strengthening emotional support during the peak period of OHSS with DVT^[8,9]^

OHSS can affect the arteriovenous system, leading to severe complications such as pulmonary embolism, myocardial infarction, or cerebrovascular accidents.^[1]^ Therefore, it is very important to pay attention to the patient’s chief complaints, examine them promptly, and actively respond. As OHSS progresses, the patient experiences worsening ascites and increased physical discomfort. Nursing interventions include actively engaging with the patient to encourage expression of their concerns and integrating health education into every interaction. In cases of abdominal distension and low back pain from severe OHSS, abdominal puncture and drainage are conducted.^[6]^ In our patient, care related to the drainage tube was implemented, and the patient’s subjective experiences post-drainage were recorded. When the patient reported soreness in both lower limbs and blurred vision, a thorough assessment was performed. VDUS confirmed increased thrombosis in the left lower limb. The nursing team guided the patient to elevate the left lower limb while encouraging ankle pump exercises using the right leg. Measurement of the leg circumference was conducted regularly, and the patient was advised to wear antithrombotic stockings for 8 to 12 hours daily until activity levels returned to baseline. Daily assessments included monitoring limb skin temperature and color, dorsalis pedis artery pulsation, and symptoms of pain or numbness.^[10]^ Detailed explanations regarding treatment and nursing aims were provided to the patient’s husband, ensuring that he was informed about disease progression, fertilization rates, and egg retrieval outcomes in a timely manner. The patient’s husband ultimately clarified his understanding and cooperated in offering emotional support for the patient together with nursing staff.

### 3.2. Regular follow-up: implementation of continuous care

#### 3.2.1. Outpatient follow-up: evaluation of patients’ medication management ability^[4,11]^

For the treatment of acute DVT, anticoagulation is still the cornerstone of treatment. For patients with acute VTE without contraindications, a 3-month anticoagulation phase is usually recommended and prolonged according to the patient ‘s individual risk factors, such as continuous stimulation conditions.^[4]^ So achieving an effective balance between anticoagulant efficacy and bleeding risk is essential for safe anticoagulant therapy and to significantly reduce thrombosis-related events.^[5]^ The patient was discharged after a 12-day hospitalization and was instructed to continue anticoagulant therapy at home, with follow-ups scheduled biweekly. Prior to discharge, the nurse reassessed the patient’s bleeding risk as well as subcutaneous injection skills and reinforced taking medication storage and usage precautions. The patient was advised to use a soft-bristled toothbrush and to avoid bumping, rubbing, or hot compresses at the injection site. After 1 month of LMWH therapy, the patient transitioned to oral rivaroxaban at a dosage of 10 mg daily. She returned to the clinic after 1 month of medication. Her nurse encouraged open communication about any concerns and provided solutions, assisting the patient in formulating personalized medication management goals. During this process, the patient reported slight, unexplained vaginal bleeding. The nurse promptly communicated this to the physician, who ruled out ovulatory and menstrual bleeding and ordered temporary suspension of anticoagulant therapy. Twelve days after discontinuation, the patient was contacted via telephone, confirming that the vaginal bleeding had stopped; then, she was encouraged to return to the clinic. Following this, the patient began taking enteric-coated aspirin at a dosage of 100 mg daily, with guidance on proper medication management.

#### 3.2.2. Shared decision-making to enhance patients’ self-management abilities

Guiding patients to reconstruct their disease narratives encourages multifaceted reflection, significantly improving their willingness to engage in decision-making and treatment compliance.^[12]^ Prior to the current treatment cycle, our patient identified intraductal papillary tumors accompanied by nipple discharge in both breasts but declined the oncologist’s recommendation for initial lesion removal, opting instead to complete her fertility plan. Understanding the patient’s personality traits, the nurse held in-depth conversations to identify her core concerns. Then, the nurse provided health education on the interplay between pregnancy hormones, tumors, and thrombosis.^[8]^ The nurse gradually introduced relevant information to the patient, offered targeted health education, and recommended appropriate. The patient’s husband was also encouraged to provide emotional support and promote her decision-making. Using narrative nursing techniques, the patient was guided to externalize, deconstruct, reframe, and reflect on her concerns.^[13]^ Finally, the patient decided to first undergo the “bilateral breast gland segment resection, bilateral breast duct selective resection (single), bilateral fascia plasty” surgery.

Sixteen months after oocyte retrieval, the patient returned to preoperatively prepare to undergo a micro-stimulation protocol for FET. Medical staff invited the patient to participate in the development of transplantation plans, and provide targeted treatment information and used teach-back skills, popular science videos, and paper information sheets to assist in health education. Posttransplantation, a follow-up plan was customized to align with the patient’s preferences. This included structured health education on weight management during pregnancy, scientific methods to protect the fetus, and regular nephrology and breast follow-up. Twenty-six days after transplantation, the patient reported multiple unexplained bruises and gingival bleeding. Blood DDi levels were measured at 0.21 µg/mL, and liver function tests returned normal results. Following the physician’s guidance, LMWH was discontinued, and the patient was started on enteric-coated aspirin at 25 mg 3 times daily. Seventy-five days posttransplant, DDi levels increased to 0.94 µg/mL, liver function and NT were normal, and the physician advised the cessation of aspirin therapy.

#### 3.2.3. Formulating a prevention and management plan for thrombosis recurrence after transplantation

ART increases the risk of VTE by 2 to 3 times, in comparison with natural pregnancy, and pregnant women have a VTE risk that is 4 to 5 times higher than that of non-pregnant women of the same age. The peak incidence of VTE occurs 3 months before pregnancy and in the postpartum period following in vitro fertilization.^[2,14]^ At the time of transplantation, our patient was a 38-year-old primipara with a history of oral contraceptive use, COH, OHSS, DVT, and unresolved renal function damage, all of which elevated her risk for recurrent thrombosis during pregnancy.^[14]^ To promote lifelong prevention of thrombosis recurrence, the nurse provided multifaceted health education. Follow-up care is a critical component of ART and includes monitoring for early pregnancy, assessing clinical pregnancy at 35 and 50 days posttransplantation, and conducting mid-pregnancy and postpartum follow-ups. Our patient anticoagulant therapy as needed during the 3 months prior to conception. After 3 months of pregnancy, the nurse worked with the patient to develop a self-management plan to help her identify ways to obtain support and assist her in being transferred to a local hospital. Additionally, telephone follow-ups were conducted at 4 months of pregnancy, 8 months of pregnancy, prior to delivery, after delivery, and 6 months postpartum to reinforce thrombosis prevention education. Nurses also used WeChat to enhance communication with the patient, promptly addressing her inquiries, providing guidance on childbirth options, and delivering comprehensive health education throughout her pregnancy.^[3,15]^

## 4. Discussion

Research indicates that approximately 25% of patients experience a relapse within 5 years following their first spontaneous VTE event, with the risk of recurrence increasing with age. The risk of 1-year recurrence in patients with a high recurrence risk is approximately 10%.^[5,14]^ The patient in this case was an older woman who had struggled with infertility for many years, and her risk of recurrent thrombosis during pregnancy persisted and increased. After a protracted and complex medical history that included OHSS, DVT, renal impairment and breast cancer, the patient was eager to have children. Through the implementation of nursing decision-making based on Orem Supportive–Educative system, evaluating patients’ self-care ability, formulating personalized goals, guiding patients to self-assess, customizing health education content, cultivating self-care skills, and gradually empowering and transferring responsibilities, early detection and early intervention can be achieved. The existing social support network can be used to provide continuous psychological counseling and empathetic communication as well. Through regular follow-up, patients can correct behavior and strengthen their autonomy. In our patient, her left lower limb swelling gradually subsided, abdominal distension discomfort was relieved, self-care ability was significantly improved, and she was able to master disease self-monitoring and nursing skills. More importantly, the patient had a successful pregnancy and birth, and mother and child were healthy. No recurrence of thrombosis occurred during pregnancy. The patient’s participation and satisfaction with nursing services were significantly improved, and she deeply understood the important role of self-care in maintaining her own health.

Currently, assisted reproductive diagnosis and treatment in mainland China are predominantly conducted in outpatient clinics. The need to improve patients’ drug management and self-care ability at home underscores the necessity for further research. This case illustrates that Orem Self-Care Theory not only offers a theoretical foundation for assisted reproductive nursing practice but can also guide nursing interventions, enhance patient engagement and satisfaction, and ultimately improve pregnancy outcomes. Furthermore, this framework opens avenues for the exploration and development of mid-range theories related to self-care among women undergoing ART.

## Acknowledgments

We thank Analisa Avila, MPH, ELS, of Liwen Bianji (Edanz) (www.liwenbianji.cn) for editing the language of a draft of this manuscript.

## Author contributions

**Formal analysis:** Aike Xu, Linfang Zhao, Qun Wei.

**Investigation:** Aike Xu.

**Methodology:** Aike Xu, Linfang Zhao, Qun Wei.

**Project administration:** Aike Xu, Qun Wei.

**Supervision:** Linfang Zhao.

**Writing – original draft:** Aike Xu.

**Writing – review & editing:** Aike Xu, Linfang Zhao, Qun Wei.

## References

[1] Leathersich S Roche C Hart R. Minimising OHSS in women with PCOS. Front Endocrinol (Lausanne). 2025;16:1507857. 40182629 10.3389/fendo.2025.1507857 PMC11966453

[2] Goualou M Noumegni S de Moreuil C. Venous thromboembolism associated with assisted reproductive technology: a systematic review and meta-analysis. Thromb Haemost. 2023;123:283–94. 36588288 10.1055/s-0042-1760255

[3] Sushko K Menezes HT Strachan P Butt M Sherifali D. Self-management education among women with pre-existing diabetes in pregnancy: a scoping review. Int J Nurs Stud. 2021;117:103883. 33548591 10.1016/j.ijnurstu.2021.103883

[4] Corvino F Giurazza F Galia M. Intravascular ultrasound findings in acute and chronic deep vein thrombosis of the lower extremities. Diagnostics (Basel). 2025;15:577. 40075824 10.3390/diagnostics15050577 PMC11898815

[5] Linnemann B Blank W Doenst T. Diagnostics and therapy of venous thrombosis and pulmonary embolism. Vasa. 2023;52(S111):1–146. 10.1024/0301-1526/a001089 40261083

[6] Bosch E Broer S; Ovarian Stimulation TEGGO. ESHRE guideline: ovarian stimulation for IVF/ICSI† [published correction appears in Hum Reprod Open. 2020 Dec 29;2020 (4):hoaa067. doi: 10.1093/hropen/hoaa067.]. Hum Reprod Open. 2020;2020:hoaa009.32395637

[7] Oh EG Lee JY Lee HJ Oh S. Effects of discharge education using teach-back methods in patients with heart failure: a randomized controlled trial. Int J Nurs Stud. 2023;140:104453. 36827745 10.1016/j.ijnurstu.2023.104453

[8] Zhu L Wang J Pan Z. Effectiveness of a family-based self-management intervention for type 2 diabetes patients receiving family doctor contract services: a community-based randomized controlled trial. J Prim Care Community Health. 2025;16:21501319251330384. 40162887 10.1177/21501319251330384 PMC11960157

[9] Jin Y Bhattarai M Kuo WC Bratzke LC. Relationship between resilience and self-care in people with chronic conditions: a systematic review and meta-analysis. J Clin Nurs. 2023;32:2041–55. 35194870 10.1111/jocn.16258

[10] Sayyadi A Mahdavi M Dalfardi B Karami Robati F Shafiepour M. Right atrial thrombus and pulmonary thromboembolism related to ovarian hyperstimulation syndrome: a case report and literature review. Clin Case Rep. 2023;11:e7018. 36911649 10.1002/ccr3.7018 PMC9992141

[11] Yang C Lee DTF Wang X Chair SY. Effects of a nurse-led medication self-management intervention on medication adherence and health outcomes in older people with multimorbidity: a randomised controlled trial. Int J Nurs Stud. 2022;134:104314. 35849886 10.1016/j.ijnurstu.2022.104314

[12] Zhang LH Meng HY Wang R Zhang YC Sun J. Application of narrative nursing in the families of children with biliary atresia: a retrospective study. World J Clin Cases. 2021;9:10557–65. 35004987 10.12998/wjcc.v9.i34.10557 PMC8686143

[13] Meng Z Liu L Yang X. High preoperative D-dimer increases the risk of venous thromboembolism after gynecological tumor surgeries: a meta-analysis of cohort studies. Res Pract Thromb Haemost. 2025;9:102690. 40236286 10.1016/j.rpth.2025.102690 PMC11999332

[14] Grouzi E Pouliakis A Aktypi Α. Pregnancy and thrombosis risk for women without a history of thrombotic events: a retrospective study of the real risks. Thromb J. 2022;20:60. 36203153 10.1186/s12959-022-00419-6 PMC9535874

[15] Tharani A Van Hecke A Ali TS Duprez V. Factors influencing nurses’ provision of self-management support for patients with chronic illnesses: a systematic mixed studies review. Int J Nurs Stud. 2021;120:103983. 34147728 10.1016/j.ijnurstu.2021.103983

